# Violaceous scaly plaques of the dorsal hands

**DOI:** 10.1016/j.jdcr.2024.05.007

**Published:** 2024-05-15

**Authors:** Rodney Ahdoot, Joanna Rew, Kelly A. Reynolds, Lori Lowe, Julie E. Mervak

**Affiliations:** a University of Michigan Medical School, Ann Arbor, Michigan; b Department of Dermatology, University of Michigan, Ann Arbor, Michigan; c Department of Pathology, University of Michigan, Ann Arbor, Michigan

**Keywords:** cutaneous side effects, dermatomyositis, dermatomyositis-like eruption, drug-induced dermatomyositis, hydroxyurea, hydroxyurea-associated nonmelanoma skin cancer, nonmelanoma skin cancer, squamous dysplasia

## Case presentation

**Fig 1:**
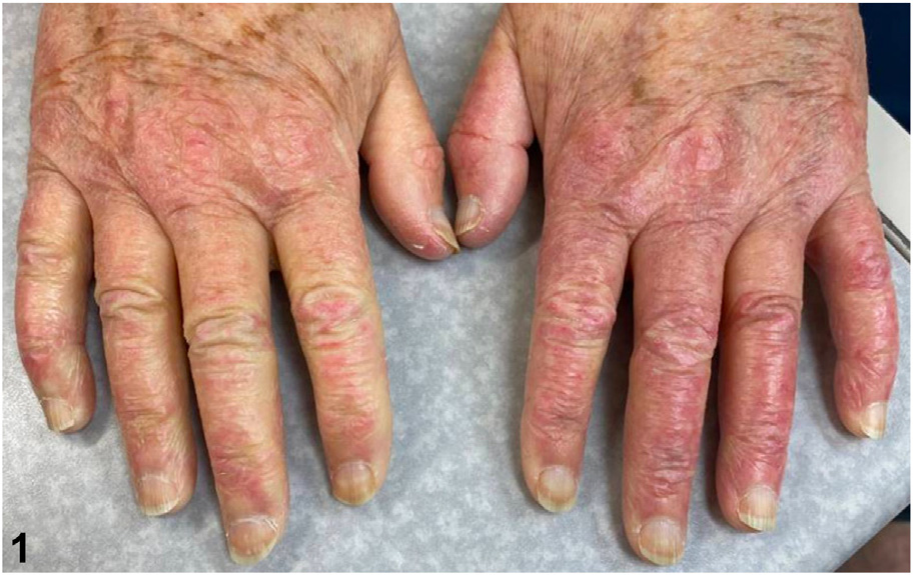

**Fig 2:**
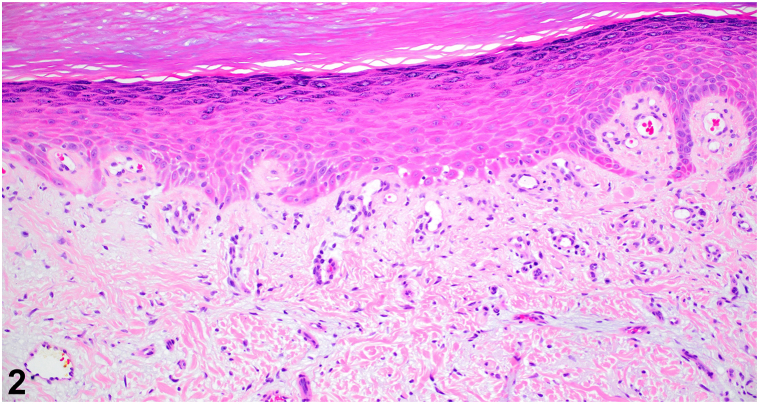

An 84-year-old woman with Janus Kinase 2 positive myeloproliferative neoplasm, managed with hydroxyurea varying between 1000 and 1500 mg by mouth daily for 12 years, presented to dermatology for evaluation of a rash on her hands. There was no improvement with emollients or triamcinolone 0.1% cream. Physical examination showed violaceous scaly, atrophic thin plaques on her dorsal hands (Fig 1). Antinuclear antibody and myositis marker panel were negative. Aldolase and creatine kinase were within normal limits. Platelet count was elevated at 584 K/uL. Biopsy revealed subtle vacuolar interface dermatitis with a mild increase in dermal mucin and vascular ectasia (Fig 2).

**Question 1: What is the most likely diagnosis?**

A. Drug-induced psoriasis
B. Acral erythema (hand-foot syndrome)
C. Hydroxyurea-induced dermatomyositis (DM)-like eruption
D. Erythromelalgia
E. Chronic actinic dermatitis

**Answers:**

A. Drug-induced psoriasis – Incorrect. Hydroxyurea is not a culprit medication for drug-induced psoriasis and can be used as a treatment for refractory psoriasis.
B. Acral erythema (hand-foot syndrome) – Incorrect. Acral erythema can result from use of hydroxyurea^1^; however, this typically presents with erythema, edema, and pain of the palms and soles, in contrast to the dorsal surface as seen in this patient.
C. Hydroxyurea-induced DM-like eruption – Correct. Hydroxyurea-induced DM-like eruption has a delayed onset after initiation of hydroxyurea, presentation ranging from 7 months to 10 years.^1^ The scaly erythematous to violaceous papules and plaques with atrophy and telangiectasias on the dorsal hands are clinically indistinguishable from true DM; however, DM-like eruption cases are often asymptomatic and lack myositis symptoms.^2^ Creatine kinase and aldolase are typically normal and antinuclear antibody is negative.^3^ On histology, DM-like eruption shows slight epidermal atrophy, vacuolar interface dermatitis, mononuclear perivascular inflammation, dermal mucin, and telangiectasias.^3^ Recognition of this diagnosis is important to prevent confusion with true DM and unnecessary immunosuppression. Discontinuation of hydroxyurea is the main intervention and DM-like eruption has been reported to resolve within 10 days to 18 months.^1^
D. Erythromelalgia – Incorrect. Although myeloproliferative disease can be associated with erythromelalgia, this presents as episodic painful flushing and edema of the distal extremities and not persistent scaly atrophic plaques as seen in this patient.
E. Chronic actinic dermatitis – Incorrect. Although this can be seen on the dorsal hands and other photo-distributed sites, there is typically spongiosis and lymphocyte exocytosis on histology.

**Question 2: Which of these manifestations is not a potential complication of hydroxyurea therapy?**

A. Fixed drug eruption
B. Melanonychia
C. Vitiligo-like depigmentation
D. Squamous cell carcinoma
E. Leg ulcers

**Answers:**

A. Fixed drug eruption – Incorrect. Cutaneous side effects have been reported to occur in 10% to 35% of patients taking hydroxyurea, including fixed drug eruption, leukocytoclastic vasculitis, alopecia, and stomatitis.^1^^,^^3^
B. Melanonychia – Incorrect. Nail abnormalities due to hydroxyurea include brittle nails, onycholysis, and melanonychia, most commonly longitudinal pattern.^4^
C. Vitiligo-like depigmentation – Correct. Hydroxyurea can result in hyperpigmentation^1^^,^^3^ but not vitiligo-like depigmentation.
D. Squamous cell carcinoma – Incorrect. Hydroxyurea-associated nonmelanoma skin cancer is the most serious complication, as these malignancies are often aggressive, leading to significant morbidity. These tumors typically occur suddenly in photo-distributed sites after an average of 6.5 years on hydroxyurea therapy.^1^ DM-like eruption, when originally described, was thought to be a benign entity; however, there is now evidence that aberrant p53 expression caused by hydroxyurea’s antimetabolite mechanism along with ultraviolet radiation leads to a chronic phototoxic response and risk for squamous dysplasia.^1^ As DM-like eruption presents earlier in the hydroxyurea course than hydroxyurea-associated nonmelanoma skin cancer, there is argument that DM-like eruption represents a premalignant precursor and therefore a reason to find alternative therapy.^1^ It is important to note that risk of hydroxyurea-associated nonmelanoma skin cancer may persist even after stopping hydroxyurea, having been shown to develop up to 4 years later.^1^
E. Leg ulcers – Incorrect. Lower extremity ulcers are a complication of hydroxyurea and have been frequently associated with patients who develop DM-like eruption.^2^

**Question 3: In contrast to hydroxyurea-induced DM-like eruption, which of the following medications has been associated with development of true DM including positive laboratory markers and myositis symptoms?**

A. Adalimumab
B. Trimethoprim-sulfamethoxazole
C. Furosemide
D. Metoprolol
E. Ustekinumab

**Answers:**

A. Adalimumab – Correct. Tumor necrosis factor alpha inhibitors such as adalimumab have been implicated in development of drug-induced DM. Other culprit medications include immune checkpoint inhibitors such as ipilimumab, pembrolizumab, or nivolumab; statins and penicillamine.^5^
B. Trimethoprim-sulfamethoxazole – Incorrect. Although implicated in cases of Stevens-Johnson syndrome/toxic epidermal necrolysis and fixed drug eruption, to our knowledge, there are no reports cases of trimethoprim-sulfamethoxazole-induced DM.
C. Furosemide – Incorrect. Phototoxic drug eruption and bullous pemphigoid have been associated with furosemide administration, but, to our knowledge, there are no reports of furosemide-induced DM.
D. Metoprolol – Incorrect. Metoprolol is associated with lichenoid dermatitis and psoriasiform eruptions, but, to our knowledge, there are no reports of metoprolol-induced DM.
E. Ustekinumab – Incorrect. In contrast to tumor necrosis factor-alpha inhibitors, to our knowledge, there are no reports of ustekinumab-induced DM.

## Conflicts of interest

None disclosed.
